# Misconceptions of Vaping Among Young Adults

**DOI:** 10.7759/cureus.38202

**Published:** 2023-04-27

**Authors:** Roei Golan, Akhil Muthigi, Armin Ghomeshi, Joshua White, Russell G Saltzman, Parris Diaz, Ranjith Ramasamy

**Affiliations:** 1 College of Medicine, Florida State University, Tallahassee, USA; 2 Desai Sethi Urology Institute, University of Miami Miller School of Medicine, Miami, USA; 3 Herbert Wertheim College of Medicine, Florida International University, Miami, USA; 4 Interdisciplinary Stem Cell Institute, University of Miami Miller School of Medicine, Miami, USA; 5 David Geffen School of Medicine, University of California, Los Angeles, USA

**Keywords:** nicotine dependence, tobacco, vaping, smoking, young adults, vaping prevention

## Abstract

Background

Vaping is growing in popularity worldwide, especially among young adults. To develop effective tobacco prevention interventions, first, there must be an understanding of the attitudes and perceptions of young adults toward the use of vaping. Highlighting perception discrepancies between races may allow physicians to more effectively counsel their patients regarding the risks of vaping.

Methodology

We conducted an online survey using Amazon Mechanical Turk (MTurk, https://www.mturk.com/) to identify misconceptions about vaping among adults aged 18 to 24 years who currently vape. The survey consisted of 18 questions evaluating reasons for vaping, history of tobacco use, and thoughts on the adverse effects of vaping. The Penn State Electronic Cigarette Dependence Index was implemented to assess dependence. Exclusion criteria comprised respondents who did not vape and were under the age of 18 or over the age of 24.

Results

A total of 1,009 responses were received with 66% identifying as male (*n *= 667) and 33% (*n *= 332) identifying as female. Sixty-nine percent of patients smoked cigarettes or used another form of tobacco previously (*n *= 692). Of those respondents, 81% indicated that they had since quit using tobacco products (excluding vaping). Switching to vaping was the most common reason for quitting cigarettes or other forms of tobacco, with health concerns and social purposes being the second and third most common reasons provided, respectively.

When asked whether vaping had negative health impacts, only 238 (24%) participants strongly agreed with this statement, while a majority (64%) neither agreed nor disagreed or only somewhat agreed. Most participants were white or Caucasian (*n *= 777). When asked whether smoking or vaping had more severe health implications, 55% of white or Caucasian participants, 41% of Asian participants, and 32% of black or African American participants indicated that vaping was worse than smoking cigarettes.

The average Penn State dependence score was 8.7, suggesting medium dependence.

Conclusions

Our survey sample of 1,006 young adults who vape indicated that the majority did not perceive vaping as significantly harmful. A comprehensive smoking prevention policy, educational interventions, and quit support are needed to enhance awareness among young adults about the health effects associated with vaping. Such interventions should also consider the novel shift toward the replacement of smoking with vaping.

## Introduction

Electronic cigarette (e-cigarette) use, commonly known as vaping, is often advertised as a safer alternative to traditional cigarettes and promoted as an aid to smoking cessation [1]. The prevalence of vaping has alarmingly risen in the last decade in the United States, and the use among young adults is particularly concerning [2]. The National Youth Tobacco Survey reports that vaping among youths has become an *epidemic* [3]. The rise in use, especially among the younger population, is partially due to the e-cigarette’s appeal as a less harmful option for tobacco smoking [1]. These products often contain artificial flavors to further appeal to a younger target audience and result in long-term use [4]. Young adults, including students in particular, are heavily influenced by their peers, and social pressure has even been documented as a primary cause for the surge in vaping [5].

While the long-term effects of e-cigarette use are not fully clear, current data report a variety of side effects associated with vaping. Pulmonary dysfunction and respiratory distress due to e-cigarette or vaping use-associated lung injury (EVALI) have become a growing concern across the country since 2018 [6]. In addition, e-cigarette users are also more likely to suffer from psychiatric conditions such as depression, anxiety, and attention-deficit/hyperactivity disorder [7,8]. E-cigarettes can damage DNA repair mechanisms and are linked to the progression of both lung and bladder cancer [9]. Additionally, several studies have found the presence of carcinogens in e-cigarette vapor, including formaldehyde, benzene, and tobacco-specific nitrosamines carcinogens [10-13].

Information on the specific attitudes and beliefs young adults possess toward vaping can provide valuable information for developing effective primordial and primary prevention programs [14]. Young adults are a vulnerable population, and it is imperative to curb the rate of vaping among the youth to prevent any potential long-term complications. There is a lack of studies that explore the attitudes and beliefs young adults have toward vaping. The objective of our study was to investigate motives and thought patterns young adults hold towards vaping. We hypothesized that young adults that vape will have prevalent discrepancies and misconceptions towards vaping.

## Materials and methods

Amazon Mechanical Turk (MTurk, https://www.mturk.com/) was used to survey young adults that vape between October 13 and October 27, 2022. The survey was available to complete for individuals aged 18 to 24 years who currently vape. Only respondents who met the age and vaping status criteria were allowed to participate in the survey, which was determined by the first two questions. Participation was voluntary and anonymous. MTurk is a crowdsourcing marketplace that allows online users to register and complete human intelligence tasks, such as online surveys, in exchange for monetary compensation. Participants were compensated $0.50 for their participation based on an estimated completion time of seven minutes. To eliminate the risk of automated computer programs, ballot-box stuffing, and task repetitions, users were required to enter their unique MTurk ID and captcha before beginning the survey [15].

Participants completed an 18-question survey that included the 10-item Penn State Electronic Cigarette Dependence Index (ECDI) to assess dependence and additional questions to assess the perception of vaping (Table 1). Informed consent was provided by each participant before completing the survey. Survey questions involved smoking history and perceptions toward vaping and smoking tobacco. Demographic information, including gender, race, and geographic location of residence, was also collected. The survey explored participants' perceptions about the potential negative health effects of vaping and their comparative beliefs about the relative harm of smoking versus vaping. The Penn State ECDI [16] includes 10 questions grading dependence (Appendix).

**Table 1:** Demographics and additional survey questions.

Demographics and questions	*N* (%)
Gender	
Male	652 (64)
Female	328 (32)
Nonbinary/third gender	3 (0.30)
Prefer not to say	6 (0.60)
Race	
White or Caucasian	777 (77)
Black or African American	69 (6.9)
American Indian/Native American or Alaska Native	35 (3.5)
Asian	107 (11)
Native Hawaiian or Other Pacific Islander	4 (0.40)
Other	12 (1.2)
Prefer not to say	5 (0.50)
Were you a previous tobacco smoker?	
No	314 (31)
Yes	692 (69)
If you were a previous tobacco smoker, have you quit?	
No	134 (19)
Yes	558 (81)
Why did you quit tobacco?	
I started vaping instead	245 (44)
It was too expensive	107 (19)
I was concerned about my health	164 (29)
Childbirth or pregnancy	39 (7)
Other: Please specify	2 (0.36)
Why do you vape?	
For social purposes	185 (18)
I like how it makes me feel	359 (36)
It is fun	136 (14)
Alternative to smoking cigarettes	280 (28)
Alternative to marijuana	35 (3.5)
Other: Please specify	6 (0.60)
How long have you been vaping?	
Under one year	87 (8.6)
One to two years	311 (31)
Two to three years	354 (35)
Three to four years	168 (17)
Four to five years	56 (5.6)
Over five years	25 (2.5)
Vaping is bad for my health.	
Strongly disagree	31 (3.1)
Somewhat disagree	91 (9)
Neither agree nor disagree	174 (17)
Somewhat agree	472 (47)
Strongly agree	238 (24)
Which do you think is worse for your health?	
Smoking	409 (41)
Vaping	513 (51)
Unsure	84 (8.4)

We employed the chi-square test to compare the proportion of Caucasian and black participants who identified that vaping was worse than smoking. The level of statistical significance was set at *α* = 0.05.

## Results

Out of the 1,009 responses, 667 (66%) were male, 332 (33%) were female, and 10 (1%) were either other, nonbinary, or preferred not to say. Racial categories included 777 (77%) white or Caucasian participants, 69 (6.8%) Black or African American participants, 107 (11%) Asian participants, 39 (3.9%) American Indian/Native American or Alaska Native participants, and 17 other (1.7%). Most participants were from the United States, while succeeding countries included India (*n *= 40), Brazil (*n *= 10), Italy (*n *= 3), and seven other countries (*n *= 8). Three hundred fourteen (31%) participants never smoked or used a form of tobacco before vaping, while 692 (69%) did. Out of the participants who did smoke or use tobacco in the past, 558 (81%) of them say they have quit (not including vaping). The top two reasons for quitting tobacco included: "I started vaping instead" (44%) and "I was concerned about my health" (30%). Most respondents indicated a vaping habit duration of ≤3 years (75%). The top reasons for vaping included: "I like how it makes me feel" (36%), "Alternative to smoking cigarettes" (28%), and "Social purposes" (18%) (Table 1).

**Figure 1:**
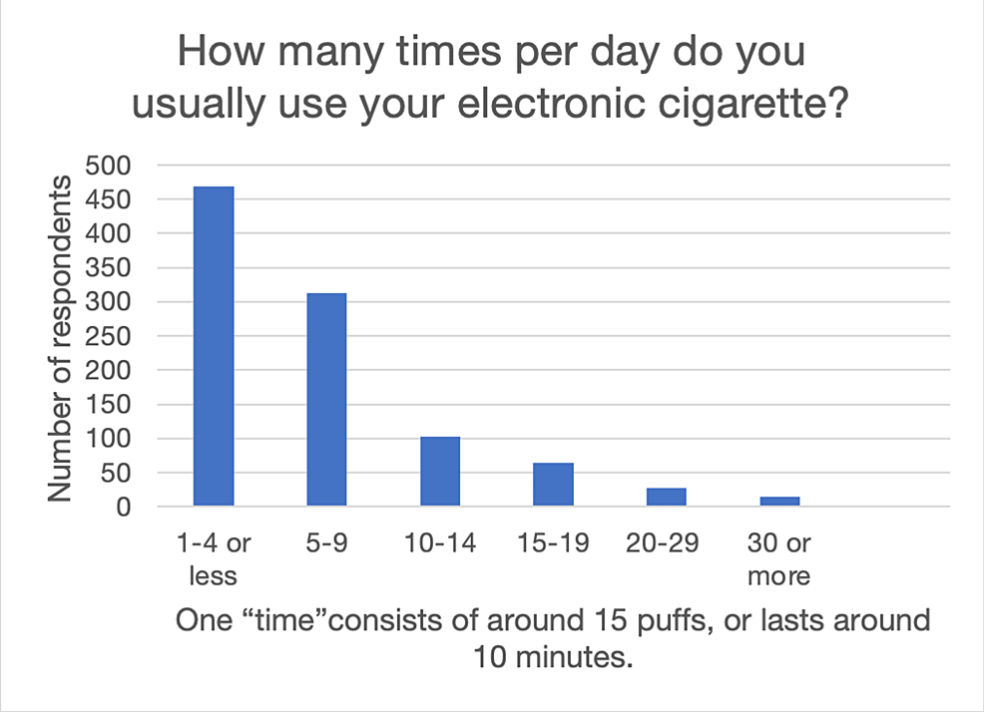
How many times per day do you usually use your electronic cigarette?

The Average Penn State dependence score was 8.7 (0-3 = not dependent; 4-8 = low dependence; 9-12 = medium dependence; 13 or more = high dependence). White participants averaged medium dependence scores (9.04), while black (8.17) and Asian (6.87) participants demonstrated low dependence scores. Data on how often respondents vape are shown in Figure 1.

When asked whether vaping was bad for their health, only 238 (24%) participants strongly agreed with this statement, while the remaining majority neither agreed nor disagreed or only somewhat agreed. When asked whether smoking or vaping was worse for their health, 41% of participants indicated smoking was worse, 51% indicated vaping was worse, and 8.2% indicated they were unsure. Four hundred twenty-five (55%) Caucasian participants, 44 (41%) Asian participants, and 22 (32%) black or African American participants indicated that vaping was worse than smoking. Statistical analysis revealed a significant difference in opinion between the Caucasian and black or African American participants (*P *< 0.05).

## Discussion

Less than a quarter of respondents strongly agreed that vaping was bad for their health, while the other 76% somewhat agreed, neither agreed nor disagreed, somewhat disagreed, or strongly disagreed. Many patients recognized that smoking tobacco was detrimental to one’s health, although we were surprised at the respondents that did not believe that vaping was detrimental. Persistent use of vaping has been associated with anxiety, depression, and suicidality [17]. Additionally, there is a risk to develop vaping-associated lung injury, not to mention the poorly understood carcinogenic potential of vaping [6,18]. Other studies have shown damage to epithelial cells in the respiratory tract, the presence of pulmonary infiltrates, and increased prevalence of chronic obstructive pulmonary disease associated with long-term vaping [19]. Our data suggest that some young adults have misconceptions about the negative impacts vaping can have on human health.

Contrary to our expectations, many respondents viewed vaping as a safer alternative to smoking tobacco, particularly non-white respondents. E-cigarettes do avoid many of the harmful combustible tobacco products such as arsenic [20], which may contribute to the perception that vaping is less harmful than smoking. Furthermore, the variety of flavors available and the attractiveness of the vaping pen designs may be seen as indicative of a safer product [4,21]. Previous studies that explored the attitudes and beliefs young adults have toward vaping confirmed that adolescents believe vaping to be not only less harmful but also less addictive [5,22,23]. Additionally, younger adults are more prone to peer pressure and tend to behave in a way they believe to be socially acceptable [5,23]. The peer pressure effect was highlighted in our study, as close to one-third of patients began vaping for the social aspect or because it was *fun*. Prior data similarly demonstrates that vaping is heavily influenced by one’s social circle and peer effect [23]. A significant portion of the younger population may simply be attempting to conform with their peers, which can act as a catalyst and progress to addictive behavior use.

We also investigated any potential racial discrepancies highlighted in the data. We found that a higher percentage of Caucasian patients identified that vaping was worse than smoking (*P *< 0.05), although the majority of patients who vape were Caucasian. Furthermore, Caucasian participants demonstrated higher dependence scores than black and Asian participants, although significance could not be evaluated in this case. Prior cross-sectional studies have shown that vaping is more common among Caucasians compared to Blacks or African Americans [24]. The increased use in this population parallels our findings suggesting higher dependence on vaping in white participants [25]. White adolescents who use e-cigarettes are also more likely to transition into cigarette smoking compared to African Americans [26]. Therefore, it is crucial to continue researching racial and ethnic factors that influence smoking behaviors. Interestingly, data on the association between socioeconomic status (SES) and e-cigarette use is limited; however, available studies have shown that lower SES is associated with greater e-cigarette use in adolescents, but the opposite trend is seen in adults [27,28]. Other studies report no association between SES and vaping [29]. More data are needed to elucidate the relationship between SES and vaping. Ultimately, targeted interventions are needed based on an individual's sociocultural background and their specific challenges.

The rapid growth in vaping is an alarming trend, as the use of e-cigarettes in the United States among young adults has continued to increase [30]. Previous research has suggested the rise in vaping is partly due to efforts to permanently quit traditional cigarette use [31]. On the other hand, past studies have reported young adults and college students do not use e-cigarettes to aid in the reduction of nicotine dependence [32,33]. Currently, there is controversy as to whether vaping is an effective means of cigarette cessation or if it will only lead to continued smoking or possibly transition into cigarette or marijuana use. Regardless, public health messaging must continue to emphasize that vaping is not a safe alternative to smoking cigarettes. It is imperative to gain a deeper understanding of the attitudes and beliefs people have toward vaping to develop targeted therapeutic strategies for cessation. Future research is needed to further investigate misconceptions younger adults and adolescents may have toward vaping. Social and cultural analyses can aid in identifying trends seen across various racial groups, allowing physicians and public health officials to develop awareness strategies starting at an early age and individualized cessation programs.

There are several limitations to consider when interpreting the results of this study. First, the use of self-report measures may have led to self-misrepresentation and social desirability bias among participants, which could affect the accuracy and reliability of the results. Second, the sample was limited to young adults who volunteered to participate in an online survey on Amazon Mechanical Turk, which may not be representative of all young adults who vape and could limit the generalizability of the results. Additionally, there is a possibility of random error and the sample size was relatively small, which may have limited the power of the statistical analyses and the ability to detect significant differences between groups. The survey used in this study also included a limited number of questions related to attitudes and beliefs toward vaping, and more in-depth and comprehensive measures may be needed to understand these attitudes and beliefs fully. Due to the limited survey design, we were unable to obtain standard deviation data and, therefore, unable to determine the statistical significance of results on dependence scores.

## Conclusions

Young adults reported utilizing vaping as a substitute for cigarette smoking, for social purposes, or simply for pleasure. Additionally, there exists a significant variance in perspectives concerning the detrimental impacts of vaping among young adults and races. These findings highlight the need for targeted interventions to educate young adults about the risks of vaping and to help them make informed decisions about their health. Further research is needed to better understand the attitudes and beliefs of young adults toward vaping and develop effective strategies for preventing and reducing vaping among this population.

## Appendices

Appendix: The Penn State Electronic Cigarette Dependence Index (ECDI)

1. How many times per day do you usually use your electronic cigarette?

2. On days that you can use your electronic cigarette freely, how soon after you wake up do you first use your electronic cigarette?

3. Do you sometimes awaken at night to use your electronic cigarette?

4. If yes, how many nights per week do you typically awaken to do so?

5. Do you use an electronic cigarette now because it is really hard to quit (using e-cigs)?

6. Do you ever have strong cravings to use an electronic cigarette?

7. Over the past week, how strong have the urges to use an electronic cigarette been?

(Check one) No urges; Slight; Moderate; Strong; Very strong; Extremely strong.

8. Is it hard to keep from using an electronic cigarette in places where you are not supposed to?

9. When you have not used an electronic cigarette for a while, OR when you tried to stop using one: Did you feel more irritable because you couldn’t use an electronic cigarette?

10. When you have not used an electronic cigarette for a while, OR when you tried to stop using one: Did you feel nervous, restless or anxious because you couldn’t use an electronic cigarette?
